# Proof of concept of a method that assesses the spread of microbial infections with spatially explicit and non-spatially explicit data

**DOI:** 10.1186/1476-072X-7-58

**Published:** 2008-11-18

**Authors:** Ariel L Rivas, Kevin L Anderson, Roberta Lyman, Stephen D Smith, Steven J Schwager

**Affiliations:** 1 Department of Population Health and Pathobiology, College of Veterinary Medicine, North Carolina State University, Raleigh, North Carolina, USA; 2 Department of Biological Statistics and Computational Biology, College of Agriculture and Life Sciences, Cornell University, Ithaca, NY, USA; 3 Institute for Resource Information Systems, College of Agriculture and Life Sciences, Cornell University, Ithaca, NY, USA

## Abstract

**Background:**

A method that assesses bacterial spatial dissemination was explored. It measures microbial genotypes (defined by electrophoretic patterns or EP), host, location (farm), interfarm Euclidean distance, and time. Its proof of concept (construct and internal validity) was evaluated using a dataset that included 113 *Staphylococcus aureus *EPs from 1126 bovine milk isolates collected on 23 farms between 1988 and 2005.

**Results:**

Construct validity was assessed by comparing results based on the interfarm Euclidean distance (a spatially explicit measure) and those produced by the (non-spatial) interfarm number of isolates reporting the same EP. The distance associated with EP spread correlated with the interfarm number of isolates/EP (*r *= .59, *P *< 0.02). Internal validity was estimated by comparing results obtained with different versions of the same indices. Concordance was observed between: (a) EP distance (estimated microbial dispersal over space) and EP speed (distance/year, *r *= .72, *P *< 0.01), and (b) the interfarm number of isolates/EP (when measured on the basis of non-repeated cow testing) and the same measure as expressed by repeated testing of the same animals (*r *= .87, *P *< 0.01). Three EPs (2.6% of all EPs) appeared to be super-spreaders: they were found in 26.75% of all isolates. Various indices differentiated local from spatially disseminated infections and, within the local type, infections suspected to be farm-related were distinguished from cow-related ones.

**Conclusion:**

Findings supported both construct and internal validity. Because 3 EPs explained 12 times more isolates than expected and at least twice as many isolates as other EPs did, false negative results associated with the remaining EPs (those erroneously identified as lacking spatial dispersal when, in fact, they disseminated spatially), if they occurred, seemed to have negligible effects. Spatial analysis of laboratory data may support disease surveillance systems by generating hypotheses on microbial dispersal ability.

## Background

Do all infections associated with the same microbial species possess similar spread ability? If not, methods that differentiate the ability of microbes to disseminate geo-temporally could lead to improved prevention or control policy.

Historically, the study of microbial spread has not considered spatially explicit (latitude and longitude) data. With the emergence of geographical information systems (GIS), the spatial dissemination of bacterial strains (genotypes) can now be explored.

The assessment of infections on the basis of microbial spread speed has been justified before: the faster the spread, the greater its dispersion [1]. To measure microbial dispersal speed (distance/time), distance needs to be estimated. GIS approaches may be used to estimate whether microbial spread is related to interfarm distances.

Surveillance systems focusing on infections involve at least 3 factors: the microbe, the host, and the environment (space). To evaluate the spread of bacterial infections, all factors of the triad need to be investigated [2-8]. Recognition of disease patterns, trends, outliers, and/or unusual events is a major focus of surveillance systems [9,10]. To diagnose and treat an infected individual, it is necessary to collect information specific to individual subjects as well as information on the population subjects belong to. Dairy farms offer an opportunity to explore the dispersion patterns of microbes across bovine populations. When new patterns or outliers are observed, pattern discovery-oriented methods may support some hypotheses of disease types more strongly than other hypotheses. Before the generalizability of pattern discovery-oriented methods is determined, its "proof of concept" (construct and internal validity) needs to be explored [11].

Here, two dimensions of the proof of concept of a method that estimates microbial diffusion were investigated. To assess construct validity, data routinely collected in clinical laboratory settings were used to ask whether all infections associated with the same microbial species are similar in their ability to disseminate geo-temporally and, if not, whether different infection types could be suggested by the data. Second, it was asked whether alternative versions of the construct (with or without inclusion of additional variables that might control for potential sources of bias) could lead to different results (internal validity).

Operationally, the null research hypothesis was that no concordance among several versions of the evaluated method would be observed, while the alternative hypothesis was that at least two indices would yield similar results (a "triangulation" approach that assesses both construct and internal validity). Support of the null hypothesis would lead to abandoning further evaluations (e.g., not to engage in explorations of the generalizability of this method), while rejection of the null hypothesis would justify future studies with the purpose of exploring external (generalizability) and, in addition, statistical validity [11].

## Materials and methods

### Collection of milk samples and identification of microbial subspecies

*Staphylococcus aureus *(n = 1126 isolates) was obtained from bovine milk samples (5–20 mL each) collected between 1988 and 2005 on 23 North Carolina dairy farms as the result of clinical work (a non-randomized dataset). *S. aureus *subspecies were characterized by pulsed field gel electrophoretic patterns (EP) [12]. Isolates were identified by: a) collection time, b) farm (latitude and longitude), c) animal, and d) EP descriptor.

### Unit of study

The unit of study was multi-dimensional, which included: 1) the host, 2) the local site (farm), 3) microbial frequency, 4) interfarm (Euclidean) distance), and 5) annual microbial diffusion speed. For the purposes of this evaluation, the population of interest was the dataset under study. Because the dataset utilized was based on materials submitted to a mastitis diagnostic laboratory, no specific conclusions can be made with respect to the epidemiology of *S. aureus *mastitis in North Carolina.

### Spatial data and Geographical Information Systems-related procedures

County-level data on dairy farms, dairy cows, and dairy farm size were obtained from the 2002 North Carolina Census of Agriculture [13]. Road data were collected from commercial archives (ESRI, Redlands, CA, USA). Farm spatial data were extracted from laboratory records.

An interfarm distance matrix (km) was created using *ArcView 3.3 *and *ArcGIS 9.2 *as reported before [14]. It included 253 interfarm distances (not shown, available upon request). The distance each EP disseminated over space, if any, was assumed to be the Euclidean distance between farm pairs when only one new infected farm was added to the dataset over a year. For instance, in 1988, EP *38 *was only found in farm *7 *but, in 1991, it was found in farm *20 *(Table 1), hence, EP *38 *was assumed to disseminate 27.25 km between 1988 and 1991 (the distance between farms *7 *and *20*, not shown). When an EP was first reported in ≥ 2 farms, later spreads were calculated as the average distance between farms. For instance, in 1988, EP *29 *was reported in 3 farms (*1*, *2*, and *7*) and in 1990, it was isolated in farms *2 *and *3*; hence, its spread was taken to be the mean distance between farms *1 *and *3*, farms *2 *and *3*, and farms *7 *and *3 *(Tables 1, 2). When an EP was later collected in ≥ 2 farms (e.g., EP *10 *in 1993), the distance was estimated as the averaged sum between the location of the farms reported earlier and the locations of farms reported later (e.g., for EP *10*, the average of the sum between the distance between farm *1 *and *3*, and that between *1 *and *7*, Tables 1 and 2).

**Table 1:** EP temporal spread among farms infected between 1988 and 2005

EP ID# (n = 23)	88	89	90	91	92	93	94	95	96	97	98	99	00	01	02	03	04	05	Mean farms/year^a^
																			
***# 2***											*2*			*2*					**0.50**
***# 5***											*2*	*2*	*2*	*2*					**1.00**
***# 7***													*10*	*1*	*1*	*3*	*3*		**1.00**
***# 10***				*1*		*1,37*		*9*		*4*		*1,2*	*1, 3*	*1*	*1*	*1*			**1.00**
***# 13***											*2*	*2*	*2,36*	*1,2, 6,7*	*1*	*1,7*	*1*		**1.86**
***# 15***						*7*		*9*			*2*	*13, 22*	*1, 2,3*	*1*	*1*				**1.00**
***# 16***												*13*	*1*	*1*	*1, 18*	*1*			**1.20**
***# 17***						*3,5*							***1***			***1***	***1***		**0.42**
***# 26***												*2*		*1*	*1*	*1*			**0.80**
***# 27***														*1*	*1*	*1*	*1*		**1.00**
***# 29***	*1, 2, 7*		*2,3*	*12*		*1, 2,5*	*12*			*8*	*2*	*2,6,22*	*1,23,6 22*	*6,7, 21*	*1, 2*	*1, 7, 17*	*1,4*	*4*	**1.72**
***# 30***	*2*					*2*						*2*	*2*	7	*1*	*1*	*1, 4*	*4*	**0.55**
***# 31***	*1*					*2,3*							*2, 3, 6*	*6*	*2*				**0.53**
***# 37***														*2*	*2*	*2,3*	*1, 2*		**1.50**
***# 38***	*7*			*20*		*7*		*7*	*7*	*8*	*2*	*2*	*2,3*	*2*		*2,3*	*2,3*	*3*	**0.89**
***# 40***	*2*		*2*															*2*	**0.17**
***# 46***													*3*			*2, 3*	*2, 6*		**1.00**
***# 53***									*14*										**1.00**
***# 58***														*6*	*6*	*6*	*6*		**1.00**
***# 62***												*22*				*3*	*3, 7*		**0.67**
***# 63***									*14*							*3*			**0.25**
***# 64***															*3*	*3*	*3*	*3*	**1.00**
***# 79***															*2*	*2,3*	*1, 4*		**1.67**

Columns indicate the year (two-digit number) each EP was reported, and the farm(s) where it was located. Numbers in italics refer to farm identifiers, not number of isolations.

^a^: Mean farms/year: number of farms where the EP was isolated/times (years when EP was isolated). For example, *EP 2 *was found in one farm (farm *2*) in the first year it was observed, not found in the following 2 years, and found again in the last year it was observed (4 years in total, with 1, 0, 0, and 1 findings, or a mean = 0.5)

**Table 2:** Cumulative and annual EP spatial spread

**EP ID** **(n = 16)**	**Farms reporting EP (farm ID)**	**Cumulative distance over 18 years (*EPdist*, km)**	**Years (between first and last isolation)**	**Annual spread velocity between first and last isolation (*EPspeed*, km/year) [C/D]**
**A**	**B**	**C**	**D**	**E**
				
*15*	*7 *to *9*, to *2*, to *13*+*22*, to *1*+*3*	965.46	9	**107.27**
*79*	*2 *to *3*, to *1*+*4*	208.01	2	**104.01**
*7*	*10 *to *1*, to *3*	389.58	4	**97.40**
*16*	*13 *to *1*, to *18*	335.03	4	**83.76**
*37*	*2 *to *3*, to *1*	175.58	3	**58.53**
*10*	*1 *to *3*+*7*, to *9*, to *4*, to *2*	643.87	12	**53.65**
*62*	*22 *to *3*, to *7*	203.23	5	**40.65**
*29*	*1*+*2*+*7*, to *3*, to *12*, to *5*, to *8*, to *6 *+*22*, to *21*, to *17*, to *4*	682.27	17	**40.13**
*46*	*3 *to *2*, to *6*	145.84	4	**36.46**
*30*	*2 *to *7*, to *1*, to *4*	517.71	17	**30.46**
*13*	*2 *to *3*+*6*, to *7*	117.95	6	**19.66**
*38*	*7 *to *20*, to *8*, to *2*, to *3*	194.35	17	**11.43**
*63*	*14 *to *3*	69.50	7	**9.93**
*17*	*3 *+*5 *to *1*	104.87	11	**9.53**
*26*	*2 *to *1*	37.36	4	**9.34**
*31*	*1 *to *2*+*3*, to *6*	113.63	14	**8.11**
*Median*		198.79	6.5	**38.30**

A: Farm identifiers.

B: Estimated route of EP dissemination, based on farm location (as reported in Table 1)

C: EP spatial spread (km) over the entire length of the study period (18 years), or *EPdist*

D: Time (years) between earliest and latest EP isolation

E: EP spread velocity (km/year), or *EPspeed*

### Non-spatial and spatial indices of spatial (inter-herd) infection dispersal

The dispersal of *S. aureus *was assessed with non-spatial and spatial measures. Non-spatial measures were those that lacked an explicit estimate of distance or speed (e.g., they were not expressed in kilometers). Non-spatial microbial diffusion was estimated as indicated by the number of isolates reporting the same EP. The *isolates/EP *relationship was expressed in percentages and calculated in two ways: (a) *interfarm isolates/EP% *([total count of isolates reporting the same EP on all farms/total count of isolates reporting any EP on all farms] × 100), and (b) *intrafarm isolates/EP% *(total count of isolates reporting the same EP on a given farm/total count of isolates reporting any EP on the same farm] × 100).

In contrast, spatial measures considered distance (km) and speed (km/year), or *EPdist *and *EPspeed*, respectively. Because spatial microbial dispersal may or may not be associated with (non-spatial) interfarm dispersal, an additional measure estimated the possible interaction between non-spatial and spatial factors: the EP geotemporal index (*EPgeotemp*, the product of *interfarm isolates/EP *and *EPspeed*). Therefore, 5 measures were used: 1) *interfarm isolates*/*EP*, 2) *intrafarm isolates/EP*; 3) *EPdist*, 4) *EPspeed*; and 5) *EPgeotemp*. In this method, the *interfarm *(or *intrafarm*)*isolates/EP *measure acted as a *de facto *gold standard: it was assumed to be accurate.

The *interfarm isolates*/*EP *measure was calculated with 3 adjustments: 1) based on either repeated testing of the same cow or non-repeated testing of the same cow (where all isolates collected from the same cow and reporting the same EP were counted only once), or *non-cow adjusted *and *cow-adjusted interfarm isolates*/*EP*, respectively, 2) multiplying the *interfarm isolates*/*EP *(in either version) by the number of farms infected per EP (*farm-adjusted interfarm isolates/EP*), and 3) multiplying the previous measure by the average number of farms infected/year (*farm-, and time-adjusted interfarm isolates/EP*). The purpose of multiplying the interfarm isolates/EP by the number of farms infected by each EP was to account for the possible bias due to a high number of isolates/EP concentrated in only one farm. The purpose of multiplying the farm-adjusted measure by the average number of farms infected/year was to control for disseminations concentrated at particular time periods.

### Non-spatial indices of local (intra-herd) infection dispersal

Local infections were assessed by simultaneously considering the percent of *intrafarm isolates/EP *and the percent of *interfarm isolates/EP*. When the *interfarm *measure was low or zero (minor or no EP spatial diffusion occurred) disease was classified as local, being labeled as "farm-related" if the *intrafarm *measure was high, or "cow-related" if the *intrafarm *measure was low.

### Descriptive quantitative analysis

While not meant to generate inferences about generalizability or statistical significance, some statistical tests were applied to assess the construct and internal validity of the method evaluated. Linearity (the Ryan-Joiner test) and correlation analysis (Pearson test) were conducted with *Minitab 15 *(Minitab, State College, PA, USA).

## Results

### Background and descriptive information

**Figure 1:**
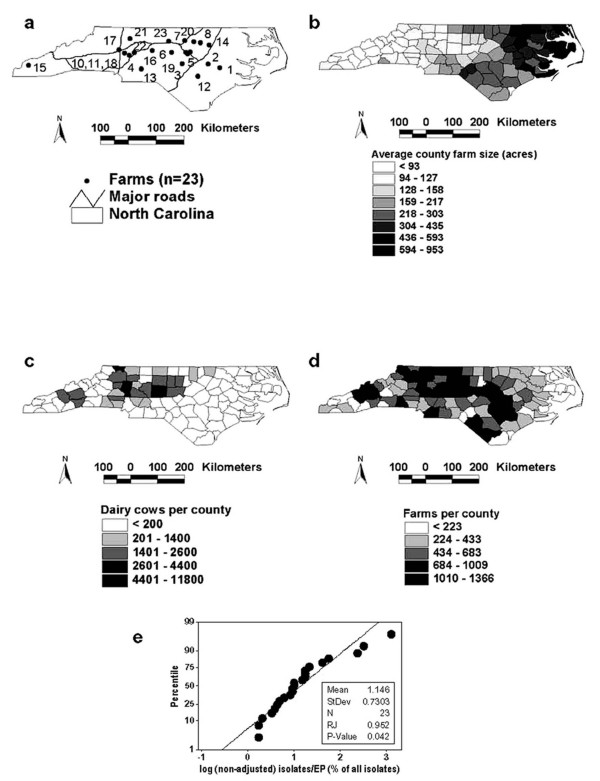
**Spatial context**. Spatial location of: investigated farms and road network (*a*), farm size (*b*), dairy cows/county (*c*), and (farms/county *d*). The Ryan-Joiner (RJ) test indicated that the (log) number of isolates/EP for the 24 microbial genotypes (EP) found in ≥ 2 isolates (interfarm EP ratio) differed significantly (*P *< 0.05) from a normal distribution (diagonal line, *e*).

The 23 investigated farms were located in counties that varied in farm size, farm density, dairy cow density, and road density (Fig. 1a–d). In those farms, 113 EPs were identified in 1126 isolates. Each EP was found, on average, 10 times (1126 isolates/113 EPs, or 9.96 isolates/EP).

Twenty-three EPs (23/113 or 20.35% of all EPs) explained 85% of all isolates (957/1126), or 4 times more cases than expected (85/20.35). Because, on average, each of these 23 EPs was found in 41.6 isolates (957/23), they were regarded to be highly frequent (HF) EPs. The remaining 90 EPs (79.6% of all EPs) were collected in 15% of all isolates (169/1126), explaining fewer cases (less than one fifth) than expected. The EPs found in only one isolate each were not analyzed. Only EPs found in ≥ 2 isolates – those with demonstrated ability to disseminate among animals- were analyzed further (n = 23 EPs).

Differences within the 23 HF EPs were also noticed in the percentage of isolates they explained: even after transformation, the (log) number of *interfarm isolates/EP *did not reveal linearity. Some EPs explained many more (less) cases than average (Fig. 1e).

By considering the number of EPs found per farm/year and the interfarm Euclidean distance, both the distance attributed to the 23 HF EPs and their speed were estimated (Tables 1, 2, 3). Only 16 of the 23 HF EPs appeared to show spatial spread (those found in ≥ 2 farms). The remaining 7 HF EPs were isolated in only one farm each (Table 1). Hence, the data indicated two infection types: with and without spatial diffusion (across- and within-herd spread, respectively).

**Table 3:** Cumulative and annual EP spread by the year 2000

EP ID*	**Cumulative EP spatial spread (*EPdist*, km)**	**Annual EP spread velocity (*EPspeed*, km/year)**
		
*15*	758.18	**108.31**
*10*	574.52	**63.83**
*29*	456.58	**38.05**
*13*	72.92	**36.46**
*38*	221.54	**18.46**
*17*	104.87	**14.98**
*31*	112.69	**9.39**
*Median*	221.54	**36.46**

*: Identifier of EPs isolated, at least, in ≥ 2 farms in ≥ 3 different years between 1988 and 2000. Both *EPdist *and *EPspeed *indicated that EP*15 *displayed values at least 3 times greater than the median.

**Figure 2:**
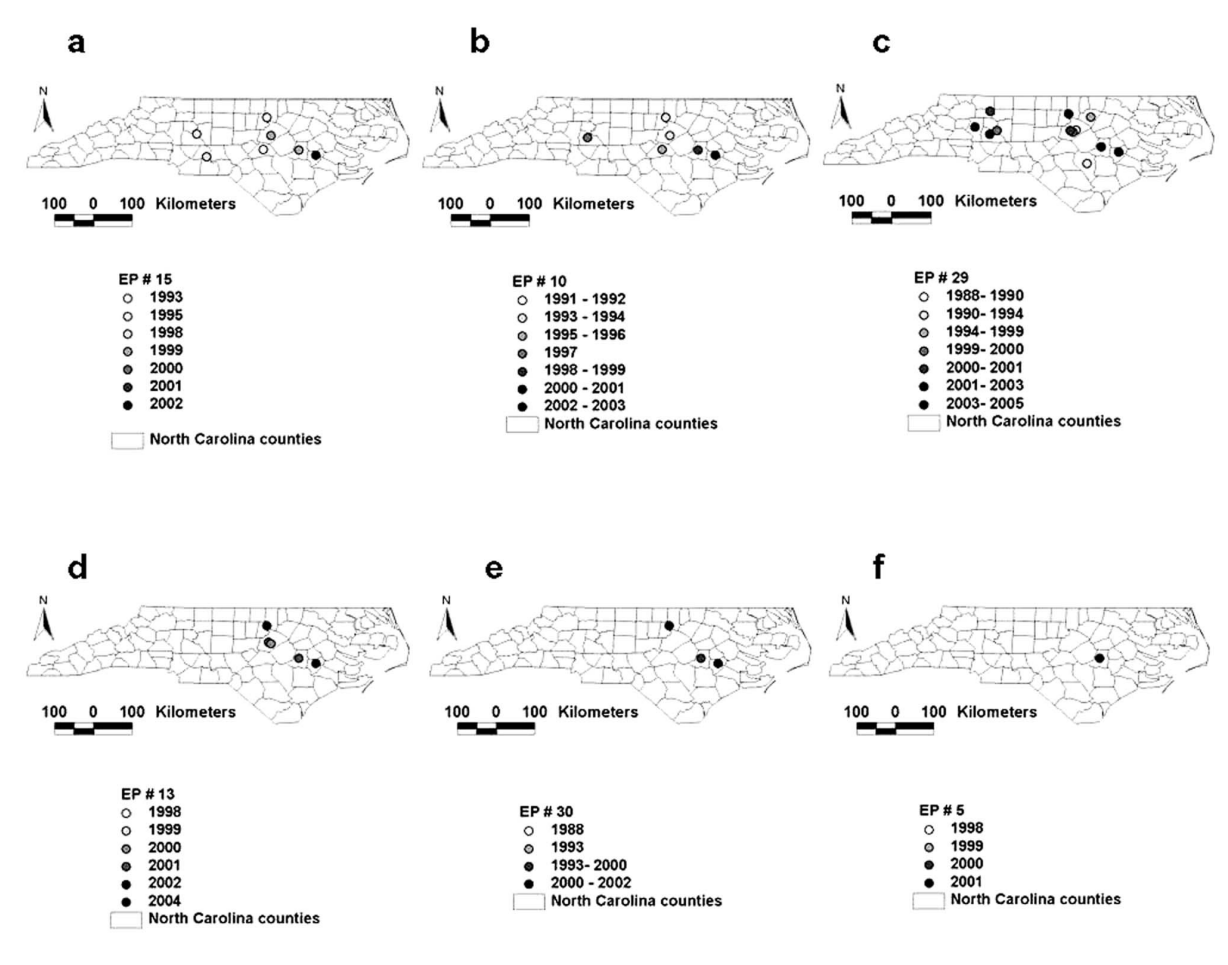
**Spatial diffusion profiles of *S. aureus *subspecies (EPs)**. High (large) spatial and high (faster) temporal diffusion (*a*, and *b*), high spatial and low (slower) temporal diffusion (*c*), low (small) spatial and low temporal diffusion (*d*), low spatial and low temporal diffusion (*e*), and local (not spatial), although frequent, diffusion (*f*). Maps display only the most recent observation on a given farm (previous observations on the same location may have occurred).

Across-herd infections revealed 4 patterns, characterized by: a) high speed and high spatial diffusion, b) low speed and low spatial diffusion, c) high speed and low spatial diffusion, and d) low speed and high spatial diffusion (Fig. 2). The data differentiated 2 sub-types within the local (within-herd) diffusion type: farm-related and animal-related infections.

### Differentiation of spatially diffused (microbial-driven or across-herd) infections

By ordering the data according to year and location (farm) each EP was reported, the EP diffusion distance (*EPdist*) was estimated. Among the 16 HF EPs that spread over space, the *EPdist *varied, within 18 years, between 37.36 and 965.46 km (Table 2, column C). When the distance between infected farms was divided by the number of years each EP was detected (the time elapsed between its earliest and latest isolation), the speed EPs spread at varied between 8.11 and 107.27 km/year (*EPspeed*, Table 2, column E).

In the spatially-disseminated infection type, neither the farm nor the cow explained disease: infection was mainly explained by highly frequent (HF) and highly spatially disseminated (HSD) EPs (i.e., those appearing to disseminate spatially above the median, Table 2). Farm *7 *was an example of a site where this infection type appeared to occur: between 1988 and 1993, it was infected by HF and HSD EPs (e.g., EPs *10, 15, 29*, Table 1).

Because not all EPs were observed in all years, *EPdist *and *EPspeed *were not always associated. For instance, EP *30 *seemed to disseminate above the median distance but below the median speed. Vice versa, EP *37 *(which showed below average *EPdist*) displayed above average *EPspeed *(Table 2, columns C, E).

Speed differences were noticed among EPs. For instance, by the year 2000, EP *15 *spread at a rate 3 times faster than the median speed of the remaining EPs (Table 3). If measures against EP *15 *had been adopted in that year (and achieved success), they could have reduced the total (cumulative) *EPdist *of EP *15 *by 21.5% (or 207.28 km, Tables 2 and 3).

Almost half of the 957 isolates where the 23 HF EPs were found, were produced by repeated testing of the same cows (Table 4, columns A-C). To prevent bias, these 23 HF EPs were also assessed on the basis of single-cow testing data (n = 485 isolates, Table 4, columns D, E).

**Table 4:** Bacterial non-spatial, spatial, and composite (non-spatial and spatial) diffusion

EP ID (n = 23)	Iso-lates (n = 957)	Iso-lates/EP (%, B/957)	(cow-adj.) Iso-lates (n = 485)	(cow-adj.) Iso-lates/EP (%, D/485)	No. of farms	(non-cow-adj.) Isolates/EP/farm (C × F)	(cow-adj.) Isolates/EP/farm (E × F)	Mean farms/year/[MFY]	(non-cow adj.) Isolates/EP/farm/MFY (G × I)	(cow-adj.) Isolates/EP/farm/MFY (H × I)	*EP dist *(km)	(non-cow adj.) EP *geo-temp *(J × L)	(cow adj.) EP *geo-temp *(K × L)
A	B	C	D	E	F	G	H	I	J	K	L	M	N
													
Nonspatial (frequency) data											Spatial	Nonspatial & spatial	
		
*29*	117	12.2	59	12.2	12	146.4	146.4	1.72	252.34	251.808	682.27	172163	171801
*10*	103	10.8	70	14.4	6	64.8	86.4	1.00	64.58	86.400	643.87	41579	55630
*15*	36	3.8	31	6.4	7	26.6	44.8	1.00	26.33	44.800	965.46	25423	43253
*79*	33	3.4	11	2.3	4	13.6	9.2	1.67	23.03	15.364	208.01	4791	3196
*13*	55	5.7	28	5.8	5	28.5	29.0	1.86	53.45	53.940	117.95	6304	6362
*38*	214	22.4	62	12.8	5	112.0	64.0	0.89	99.51	56.960	194.35	19340	11070
*16*	26	2.7	14	2.9	3	8.1	8.7	1.20	9.78	10.440	335.03	3277	3498
*46*	21	2.2	12	2.5	3	6.6	7.5	1.00	6.58	7.500	145.84	960	1094
*30*	24	2.5	16	3.3	4	10.0	13.2	0.55	5.52	7.260	517.71	2856	3759
*37*	13	1.4	7	1.4	3	4.2	4.2	1.50	6.11	6.300	175.58	1073	1106
*7*	25	2.6	6	1.2	3	7.8	3.6	1.00	7.84	3.600	389.58	3053	1402
*62*	12	1.3	6	1.2	3	3.9	3.6	0.67	2.52	2.412	203.23	512	490
*17*	48	5.0	14	2.9	3	15.0	8.7	0.42	6.32	3.654	104.87	663	383
*31*	18	1.9	9	1.9	4	7.6	7.6	0.53	3.99	4.028	113.63	453	458
*26*	26	2.7	17	3.5	2	5.4	7.0	0.8	4.35	5.600	37.36	162	209
*63*	12	1.3	11	2.3	2	2.6	4.6	0.25	0.63	1.150	69.50	44	80
*53*	33	3.4	33	6.8	1	3.4	6.8	1.00	3.45	6.800	0.00	0	0
*2*	33	3.4	31	6.4	1	3.4	6.4	0.5	1.72	3.200	0.00	0	0
*5*	16	1.7	14	2.9	1	1.7	2.9	1.00	1.67	2.900	0.00	0	0
*40*	19	2.0	11	2.3	1	2.0	2.3	0.17	0.34	0.391	0.00	0	0
*64*	31	3.2	10	2.1	1	3.2	2.1	1.00	3.24	2.100	0.00	0	0
*27*	25	2.6	9	1.9	1	2.6	1.9	1.00	2.61	1.900	0.00	0	0
*58*	17	1.8	4	0.8	1	1.8	0.8	1.00	1.78	0.800	0.00	0	0

A: EP identifier (of EPs found in ≥ 2 isolates, accounting for ≥ 1% of all isolates)

B: Isolates/EP (repeated-cow testing, total number of isolates [n = 957])

C: Percentage of isolates/EP (repeated-cow testing, percentage of all isolates ["C"= "B"/957 × 100])

D: Isolates/EP (single-cow testing, total number of isolates, [n = 485])

E: Percentage of isolates/EP (single-cow testing, percentage of all isolates ["E"= "D"/485 × 100])

F: Number of farms where a given EP was found

G: Number of isolates/EP (repeated-cow testing, multiplied by number of farms ["G"= "C" × "F"])

H: Number of isolates/EP (single-cow testing, multiplied by number of farms ["H"= "E" × "F"])

I: Mean number of farms/year in the year(s) the EP was found (or MFY, same as reported in Table 1)

J: Number of isolates/EP (repeated-cow testing, times farm/EP, and farms/year ["J"= "G" × "I"])

K: Number of isolates/EP (single-cow testing), times farm/EP, and farms/year ["K"= "H" × "I"])

L: *EPdist *(km, as reported in Table 2, column C)

M: *EPgeo-temp *index (repeated-cow testing based ["M"="J" × "L"])

N: *EPgeo-temp *index (single-cow testing based ["N"="K" × "L"])

The multiplication of the *interfarm isolates/EP *by the number of farms where each EP was found produced a measure that reflected spread beyond the boundaries of a given farm (although it did not assess distance): the (farm-adjusted) number of interfarm isolates reporting the same EP (Table 4, columns F-H). That measure was further multiplied by the mean number of sites infected per year by a given EP (a non-spatial measure that addressed the effect of time, Table 4, columns I-K). By multiplying that value by EP distance (a spatially explicit measure), the *EP geo-temporal index *(*EPgeotemp*) was obtained (Table 4, columns M, N).

**Figure 3:**
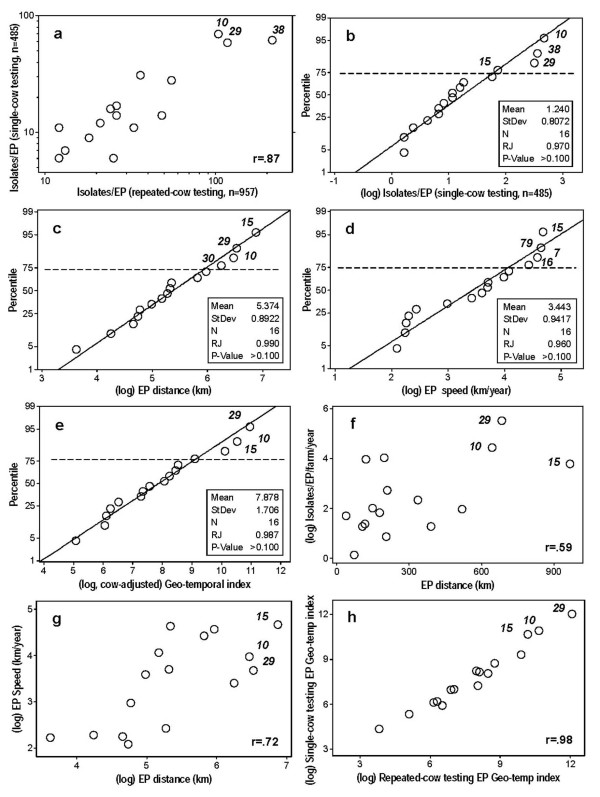
**Non-spatial and composite (non-spatial and spatial) microbial diffusion**. The number of EP-specific interfarm isolates correlated significantly between repeated and single testing of the same animals (r = .87, *P *< 0.01, as indicated in Table 4, columns B and D, *a*). Four indices that estimate bacterial diffusion are described: the (single-cow testing based) number of *isolates/EP *(as indicated in Table 4, column E, *b*), the distance assumed each EP disseminated over time (*EP distance*, as indicated in Table 2, column C, *c*); the EP diffusion velocity (*EPspeed*, as indicated in Table 2, column E, *d*); and the product of (fully adjusted) interfarm isolates/EP and EP speed (the fully adjusted geo-temporal or *EPGeotemp *index, as indicated in Table 4, column N, *e*). Diagonal lines indicate the expected distribution under the hypothesis of linearity. The null hypothesis of lack of normality was rejected (Ryan-Joiner [RJ] test > 0.05). Significant correlations were observed (i) between the *isolates/EP *index (adjusted for single-cow testing, number of farms, and time) and *EPdist *(*r *= .59, *P *< 0.02, as indicated in Table 4, columns J and L, *f*), (ii) between *EPdist *and *EPspeed *(*r *= .72, *P *< 0.01, as indicated in Table 2, columns C and E, *g*), and (iii) between both versions of the *EPGeotemp *index (non-repeated vs. repeated testing of the same cow, *r *= .98, *P *= 0.01, as indicated in Table 4, columns M and N, *h*). Numbers in italics identify EPs above the 75^th ^percentile or displaying the highest values.

The two measures of the *interfarm isolates/EP *index (the repeated-cow testing and the single-cow testing versions) correlated (r = .87, *P *< 0.01, Fig. 3a). To determine the spatial diffusion associated with each EP, the values of the 16 EPs that revealed spatial diffusion were log-transformed. Several indices identified 3 EPs (*10*, *15 *and *29*) as disseminating geo-temporally above the 75^th ^percentile (Figs. 3b–e). This means that 75% of all EPs explained fewer isolates than EPs *10*, *15 *and *29*. These 3 EPs (2.6% of all EPs) explained 26.75% of the non-adjusted (repeated cow-testing based) interfarm isolates (10 times more cases than expected, Table 4, column C), or 32.98% of all the adjusted (single-cow testing based) interfarm isolates (12 times more cases than expected, Table 4, column E). Hence, EPs *10*, *15*, and *29 *were estimated to be super-spreaders: they showed both high frequency and high geo-spatial dissemination, revealing values at least twice higher than other spatially disseminated EPs.

The spatially explicit *EPdist *(the construct under analysis) was significantly associated with the non-spatial measure of microbial dispersal (the variable regarded to be the gold standard): the (single cow-, farm-, and time-adjusted) *interfarm isolates/EP *correlated with *EPdist *(r = .59, *P *< 0.02, Fig. 3f). Two indications of internal validity were observed: 1) *EPspeed *correlated with *EPdis*t (*r *= .72, *P *< 0.01, Fig. 3g); and 2) the *EPgeotemp *index calculated on the basis of repeated testing of the same cow correlated with the index obtained in single-cow testing (r = .98, *P *< 0.01, Fig. 3h).

### Differentiation of locally diffused (not spatially disseminated or within-herd) infections

**Figure 4:**
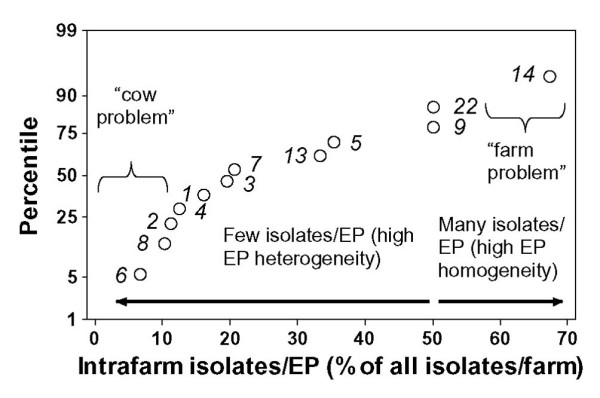
**Local (non-spatially disseminated) infection hypotheses**. The 12 farms that contributed with ≥ 6 isolates were assessed in terms of *intrafarm *isolates/EP (percentage of isolates of a given farm reporting the same EP). Numbers identify farms (the same as reported in Table 5). Because the farm displayed in the upper right quadrant showed a high intrafarm isolates/EP index (67% of all isolates collected in farm *14 *pertained to the same EP) but no spatial diffusion (zero *EPspeed*, see Tables 4 and 5), a "problem farm" hypothesis was supported. In contrast, no EP predominated in the 3 farms identified in the lower left quadrant (*6*, *8*, *2*): EPs collected in those farms explained < 13% of all isolates and displayed no spatial diffusion, profile that supported an "animal-problem" hypothesis.

The *intrafarm isolates/EP *supported a "farm-related" infection hypothesis when it was high (when > 50% of the isolates found in a farm reported the same EP) and, in addition, the *EPgeotemp index *was low or zero (when poor or no spatial diffusion was observed). "Farm-related" infections were suspected in farm *14 *(Fig. 4), where EPs *53 *was found in 67% of the isolates (Table 5), but zero *EPdist *was observed and, therefore, no *EPspeed *(no across-herd dispersion) occurred.

**Table 5:** Local (non-spatial) analysis: intrafarm and interfarm isolates/EP (%) and EP speed

**Farm ID**	**Farm iso-lates**	**Farm isolates in cows tested once**	**EPs per farm**	**MF iso-lates/EP**	**MF isolates/EP/cows tested once**	***Intra-farm *isolates/EP (%)**	**Adjusted *intrafarm *isolates/EP (%)**	**MF EP (ID)**	***Interfarm *isolates/EP%**
**A**	**B**	**C**	**D**	**E**	**F**	**G**	**H**	**I**	**J**
									
*14*	49	49	5	33	33	67.34	**67.3**	*53*	zero
*9*	6	5	3	3	3	50.0	**50.0**	*10*	high
*22*	6	6	3	3	3	50.0	**50.0**	*29*	high
*5*	17	15	6	7	6	41.18	**35.3**	*60*	low
*13*	6	6	4	2	2 and 2	33.33	**33.3**	*16 *and *42*	Average, low
*7*	29	28	16	6	6	20.69	**20.6**	*29*	high
*3*	302	126	25	181	59	59.9	**19.5**	*38*	high
*1*	362	194	38	90	58	24.8	**16.02**	*10*	high
*4*	58	19	8	26	9	44.82	**15.5**	*79*	high
*2*	240	167	34	33	33	13.7	**12.5**	*2*	zero
*8*	9	9	9	1	1	11.11	**11.1**	*29, 38, 56, 57, 67, 69, 71, 72, 76*	high, low
*6*	60	21	15	17 and 17	4 and 4	28.33	**6.7**	*29 *and *58*	high, zero

A: Farm identifier

B: Total number of isolates collected in the farm

C: Total numbers of isolates collected from different cows of the farm

D: Total number of EPs found in the farm

E: Number of isolates of the EP most frequently (MF) found in the farm

F: Number of isolates of the MF EP found in cows tested once in the farm

G: Percent of all farm isolates reporting the MF EP ("G"= "E"/"B")

H: Percent of all farm isolates reporting the MF EP, based on non-repeated cow testing ("H"="F"/"B")

I: Identifier of the MF EP isolated in the farm

J: Low, average, high: below the 50^th ^(low) or 75^th ^(average) percentile, or above the 75^th ^percentile, respectively, as reported in Table 4 and Fig. 3(b-e).

In contrast, "cow-related" infections were suggested when the *EPgeotemp index *was average or low, and the percentage of *intrafarm isolates/EP *was low (many EPs were found within a farm, so no individual EP represented > 50% of all isolates reported in the farm). "Cow-related" disease was suspected in farms *2*, *8*, and *6 *(a set representing 25% of all farms with ≥ 6 isolates, Table 5 and Fig. 4). For instance, the EP most frequently isolated in farm *2 *(EP *2*) had a low percentage of *intrafarm isolates/EP *(12.5%) and no spatial diffusion (zero *EPgeotemp*, Tables 4 and 5).

Although affecting different cows, some farms revealed ≥ 2 infection types. For instance, a double profile appeared to occur in farm *13*, where the two most frequently isolated EPs (*16 *and *42*) showed high and low *EPspeed*, respectively (Table 5). A triple profile was shown in farm *2*, where (a) an EP lacking spatial spread (EP *5*) was observed in four consecutive years (suggesting a "farm-problem" type, Table 1), (b) the EP most frequently isolated (EP *2*) revealed a low percentage of *intrafarm isolates/EP *and no spatial spread, indicating a "cow-related" problem (Tables 4 and 5), and (c) EPs with high *EPgeotemp *indices (*10*, *15*, *29*, and *79*) were also observed (Table 4).

## Discussion

This study explored whether the use of multi-dimensional indices (which included spatially explicit data) could differentiate infections in terms of their ability to disseminate across subjects, space, and time. Because test validation requires the assessment of construct, internal, external, and statistical dimensions, this report focused on the "proof of concept" (the first two dimensions). Unless construct and internal validity are defensible, the evaluation of the remaining dimensions may not be justified [11]. Because only one dataset was assessed, no conclusions could be made about (a) the generalizability of the findings, and (b) the statistical model appropriate for this method.

While transmission and contagiousness are separate concepts (e.g., one microbe may be highly contagious within a farm but not isolated across farms, while another microbe may be found in many places and times but only infecting a marginal proportion of animals), the scenario under analysis supported the hypothesis that transmission and contagiousness tend to be associated. Regardless of the (unknown) mechanism of dissemination or transmission, 3 EPs explained a higher percentage of isolates, revealing higher values of spatial-temporal dissemination than the remaining EPs.

While the data supported the overall hypothesis that infections can be differentiated on the basis of their ability to disseminate, the major issue of interest was whether the basic measure estimated by the method under analysis (which includes the use of interfarm Euclidean distance as estimator of microbial diffusion) possessed construct validity. Unknown and/or unmeasured factors, taking place over 18 years, could have influenced the observed findings, generating false negative and/or positive results (underestimating and/or overestimating the actual microbial diffusion). Hence, two questions were asked to address construct and internal validity issues: 1) did all EPs explain a similar percentage of isolates?, and 2) did the non-spatial and spatial diffusion measures correlate?

The first question was answered negatively. The second question was answered positively. The high correlation observed between the repeated- and the non-repeated isolates/EP data provided an additional evidence of internal validity (*r *= .87, Fig. 3a).

Several indices identified 3 EPs (*10*, *15*, and *29*, Figs. 3a–f) as highly spatially diffused (HSD) and highly frequent (HF). These EPs explained at least twice as many cases as explained by other EPs. That finding was not unexpected: Woolhouse's "20:80" rule has been reported in many infectious diseases. That rule indicates that a minor proportion of "cases" (in this scenario, a minor percent of EPs) explains most of the disease diffusion (epidemic) process [15-17].

These findings were relevant to assess the validity of this method: they suggested that what mattered was not whether the (interfarm Euclidean distance-based) spatial measure under study exactly reflected the true distances and/or speeds associated with the dissemination of *S. aureus*-mediated intramammary infections. Instead, the question of interest was whether the created (spatial) construct could adequately identify the EPs that contributed most to disease spread. Due to the "20:80" rule, the errors that may actually matter are those that involve the EPs displaying ultra-high frequency and/or spatial dissemination. No discrepancies were observed in relation to EPs *10*, *15*, and *29*. If some EPs were erroneously assumed to either lack spatial dissemination when in fact they spread spatially, or dispersed more than they actually did but disseminated below the 75 percentile, those errors would have negligible impact: the EPs that disseminated spatially below the 75 percentile only explained a minor percentage of isolates (they appeared to possess a marginal contagiousness).

The same EPs contributing most to infection dispersal were detected by both the non-spatial and spatial measures. Because the spatially explicit diffusion measure (*EPdist*) correlated with the non-spatially explicit diffusion measure (*interfarm isolates/EP*), errors due to actual spatial dissemination routes and/or speeds different from estimated were assumed to be inconsequential.

The "triangulation" approach followed in this method may expand the information available to decision-makers, facilitating their ability to detect inconsistencies. For example, EP *38 *was identified as highly disseminated by the (single cow-based) *interfarm isolates/EP *measure (Fig. 3b), but it was not so identified when spatially explicit measures were considered (Figs. 3c–e).

Future versions of this method could include additional variables, not assessed here or only partially described (Fig. 1a–d). For instance, disease spread could be expressed by the number of new farms infected per new year. Other measures to consider include: a) non-Euclidean distances ("along roads" interfarm distance), as described elsewhere (18); b) proximity to major highways; c) contact tracing (contacts between farms and/or between farms and markets); d) regional traffic (e.g., the influence of human and animal population density [19]); e) farm management practices; and f) immunity [12,18,20]. For example, the data shown in Figs. 1a–d could, in future assessments, investigate the association between highly frequent EPs and factors that may facilitate or prevent microbial dispersal (e.g., high vs. low road/farm/animal density, as reported elsewhere [21]).

This method addressed two needs so indicated by the disease surveillance literature: 1) the relative lack of multivariate indicators, and 2) the production of spatial disease maps. Multivariate indicators are expected to describe disease spread more validly than univariate indicators [22,23]. Spatial disease maps promote the generation of location-specific decisions [24].

The data supported two hypotheses of local (within-herd) infection. A local disease profile suggests that some (unknown) interaction may occur between the bacterial agent and local actors such that, EPs that in other farms seem to be mildly pathogenic, may induce infections. Local infection types were found within one third of all farms contributing with 8805; 6 isolates. When the EP spatial spread was poor and the percent of *intrafarm isolates/EP *was above average (when high EP homogeneity was observed within a farm, and no spatial diffusion was noticed), a "problem farm" was suspected [25]. When an EP was found in only one farm (e.g., if *EPgeotemp *was zero), the hypothesis of a "problem farm" became even stronger, as observed in farm *14 *(Tables 1 and 4).

The second local infection type ("cow-related") was suggested when the percent of *intrafarm isolates/EP *was low (many EPs were observed within a farm) and the infecting EPs showed poor or no spatial diffusion. In this type, disease was suspected to be neither microbial-driven nor farm-related but cow-related [26]. This profile was displayed by farms *2*, *6*, and *8*, which showed high intrafarm EP heterogeneity (many EPs were observed within each farm) and poor spatial spread.

Data on repeated isolations of the same EP from the same cow may be valuable in some instances. When the same EP is repeatedly isolated from the same cow and it neither reveals a high spatial diffusion nor it predominates in the farm (there is no evidence of either a microbial-driven or a farm-related problem), the host may be suspected to be the problem (e.g., an immune-related problem may be hypothesized).

The presence of mixed infection types in some farms indicated that some herds were not homogeneous. This finding documented that epidemiologic models based on the theory of homogenous population mixing may be invalid [27].

Differentiation of infection types, based on their diffusion ability, may lead to type-specific inquiries. When a farm-related infection is suspected (noticed in farm *14*), inquiries focusing on management, water quality, or local climate may be warranted. When "cow-related" infection is suspected, investigations on the immune response, nutrition, and/or genetics may be indicated [28].

The indices here described could support epidemiological inquiries (Additional file 1). They could also be applied to detect bacterial epidemics of "slow" diffusion that, otherwise, could remain undetected. False negative results (e.g., no discrimination among infection types and sub-types) and/or delayed detection of changes in disease patterns may occur when the individual scale (a single animal) is considered. However, when the population is evaluated over time and space, changes in infection diffusion patterns may become noticeable at earlier times.

Given the high percentage of isolates explained by highly spatially disseminated (HSD) and highly frequent (HF) EPs, even a minor success rate in noticing such infection type could substantially reduce the overall number of cases. If, in this scenario, only one of the 3 EPs showing the largest dispersal had been identified early (and successfully treated), between 6 and 14 percentage points of disease occurrence could have been prevented. Provided that other criteria (e.g., data quality, frequent testing) are also met, if assessed prospectively, the use of geo-temporal data could result in early detection of disease pattern changes, facilitating less costly or more beneficial decisions [9,10,22-24,29,30].

## Conclusion

Hypotheses on infection categories, classified on the basis of geo-temporal dissemination, were generated by the method under analysis. Because the interfarm Euclidian distance correlated with the isolates/EP measure and the estimated EP distance correlated with the estimated EP speed, the method seemed to possess both construct and internal validity. Further studies are recommended to explore external and statistical validity issues.

While the subjects investigated in this study were domestic animals, the methodology here described and evaluated is potentially applicable in human medicine. Two reasons support the previous statement: 1) the variables analyzed are routinely collected or potentially collected by diagnostic laboratories of human infectious diseases; and 2) while the bacterial strains analyzed in this study do not seem to infect humans, the bacterial species here assessed is a major pathogen affecting humans. While the construct and internal validities of the model evaluated could have been explored with a simulated geo-temporal dataset, we chose to assess an actual scenario, which happened to contain data from domestic animals. That selection, however, may have an advantage over either simulated studies or studies conducted with human populations: domestic animal populations are usually less prone to outside interactions (animal populations, such as herds, are relatively closed and, therefore, more apt to investigate within- and between-population factors than human populations). Hence, what here is described as a farm or herd can be considered to represent a relatively closed human environment.

## Competing interests

The authors declare that they have no competing interests. ALR and SJS are coauthors of a patent in process that measures interfarm distances as here described.

## Authors' contributions

ALR conceived the study, performed the analysis, and drafted the manuscript. KLA and RL carried out the microbiological studies and provided farm geo-referenced data. SJS created the method used to calculate interfarm distances. SDS produced the geo-referenced dataset and calculated the interfarm distances. All authors read and approved the final manuscript.

## Supplementary Material

Additional file 1 **Examples of data-driven questions, further inquiries, and possible decisions**. Click here for file
