# Supplementary material for: Proof of concept of a method that assesses the spread of microbial infections with spatially explicit and non-spatially explicit data
Source: Int J Health Geogr. 2008 Nov 18;7:58. doi: 10.1186/1476-072X-7-58 (PMC2613142; doi:10.1186/1476-072X-7-58)
Supplement: Additional file 1 — Examples of data-driven questions, further inquiries, and possible decisions. [file 1476-072X-7-58-S1.doc]

**Examples of data-driven questions, further inquiries, and possible decisions**

- Found in ≥ 2 isolates? * a contagious microbe ?
- Found in 1 isolate? * a non-contagious microbe ?
- Found in ≥ 2 sites? * spatial (inter-herd) dissemination ?
- Found in 1 site? * local (intra-herd) dissemination ?
- Spatial and non-linear distribution? * superspreaders may be present
- Spatial and above the 75 percentile? * a possible superspreader
- Local and high EP homogeneity? * farm-related ?
- Local and low EP homogeneity? * animal-related ?
- Strain-related, spatial dissemination? * strain-specific control (e.g., vaccine, isolation,

culling)

- Farm-related, local dissemination? * management, water quality, trade patterns
- Animal-related, local dissemination? * immunity, nutrition, genetics

Alternative A. Indices: Above average *EPgeo-temp* (high interfarm *EP ratio* and high *EPspeed*)

Hypothesis: Spatial (EP-specific) disease profile.

Example: Farm *7* (infected by EPs *10*, *15*, *29* and *79*)

Possible decision 🡪 EP-specific control measures

Alternative B. Indices: High intrafarm *EP ratio* and average/low *EPspeed*

Hypothesis: Local, farm-related disease profile.

Example: Farm *14*

Possible decision 🡪 inquiries: water quality, management, etc.

Alternative C. Indices: Low intrafarm *EP ratio* (high EP heterogeneity) and average/low *EPspeed*

Hypothesis: Local, cow-related disease profile.

Example: Farm *2*

Possible decision 🡪 inquiries: immunity, genetics, nutrition

Alternative D. Indices: Average *EPgeo-temp* (due to low, average or high *EP ratio* and/or *EPspeed*)

Hypothesis: Mixed disease profile.

Example: Farm *13*

Possible decisions 🡪 *sub-population specific* inquiries:

immunity, genetics, nutrition, management, and

EP-specific control measures.
